# Prostaglandin E_2_ EP2 Receptor Deletion Attenuates Intracerebral Hemorrhage-Induced Brain Injury and Improves Functional Recovery

**DOI:** 10.1177/1759091415578713

**Published:** 2015-04-07

**Authors:** Jenna L. Leclerc, Andrew S. Lampert, Matthew A. Diller, Joshua B. Immergluck, Sylvain Doré

**Affiliations:** 1 Department of Anesthesiology, University of Florida, Gainesville, FL, USA; 2 Department of Neuroscience, University of Florida, Gainesville, FL, USA; 3 Departments of Neurology, Psychiatry, and Pharmaceutics, University of Florida, Gainesville, FL, USA

**Keywords:** collagenase, gliosis, iron, neuroinflammation, neuroprotection, stroke

## Abstract

Intracerebral hemorrhage (ICH) is a devastating type of stroke characterized by bleeding into the brain parenchyma and secondary brain injury resulting from strong neuroinflammatory responses to blood components. Production of prostaglandin E_2_ (PGE_2_) is significantly upregulated following ICH and contributes to this inflammatory response in part through its E prostanoid receptor subtype 2 (EP2). Signaling through the EP2 receptor has been shown to affect outcomes of many acute and chronic neurological disorders; although, not yet explored in the context of ICH. Wildtype (WT) and EP2 receptor knockout (EP2^−/−^) mice were subjected to ICH, and various anatomical and functional outcomes were assessed by histology and neurobehavioral testing, respectively. When compared with age-matched WT controls, EP2^−/−^ mice had 41.9 ± 4.7% smaller ICH-induced brain lesions and displayed significantly less ipsilateral hemispheric enlargement and incidence of intraventricular hemorrhage. Anatomical outcomes correlated with improved functional recovery as identified by neurological deficit scoring. Histological staining was performed to begin investigating the mechanisms involved in EP2-mediated neurotoxicity after ICH. EP2^−/−^ mice exhibited 45.5 ± 5.8% and 41.4 ± 8.1% less blood and ferric iron accumulation, respectively. Furthermore, significantly less striatal and cortical microgliosis, striatal and cortical astrogliosis, blood–brain barrier breakdown, and peripheral neutrophil infiltration were seen in EP2^−/−^ mice. This study is the first to suggest a deleterious role for the PGE_2_-EP2 signaling axis in modulating brain injury, inflammation, and functional recovery following ICH. Targeting the EP2 G protein-coupled receptor may represent a new therapeutic avenue for the treatment of hemorrhagic stroke.

## Introduction

Among the various types of stroke, intracerebral hemorrhage (ICH) is one of the most disabling and has the highest mortality rates (van Asch et al., 2010; Gonzalez-Perez et al., 2013). Further, there are currently no effective therapies for the treatment of ICH, and the clinical management of patients focuses on supportive measures as surgical and medical management approaches have failed to improve outcomes (Sahni and Weinberger, 2007; Mayer et al., 2008; Rincon and Mayer, 2008; Crandall et al., 2011). Primary injury after ICH results from the influx of blood into the brain parenchyma leading to mass effects, increased local pressure, and disruption of brain architecture (Aronowski and Zhao, 2011; Mendez et al., 2013). Secondary damage is a chronic process attributable to the cytotoxicity and resulting strong inflammatory responses initiated by the presence of blood components and is the likely culprit of pervasive, and often irreversible, neurological deficits (Chen and Regan, 2007; Wang and Doré, 2007b; Aronowski and Zhao, 2011). Understanding the pathways involved during these secondary processes may provide additional insight into ICH pathophysiology and uncover new therapeutic targets.

Neuroinflammatory insults lead to increased production of prostaglandin E_2_ (PGE_2_), a potent biolipid messenger that plays an important role in many physiological and pathological conditions in the brain (Jiang and Dingledine, 2013; Johansson et al., 2013). PGE_2_ levels are increased in response to inflammation by cyclooxygenase-2 (COX-2), the inducible form of cyclooxygenase, and a rate-limiting enzyme responsible for formation of prostaglandins and thromboxane from arachidonic acid (Candelario-Jalil et al., 2005; Ahmad et al., 2006b). PGE_2_ has many diverse actions, with toxic and neuroprotective effects, as a result of differential activation of mainly four E prostanoid (EP) receptors, EP1-4 (Manabe et al., 2004; Pooler et al., 2004; Echeverria et al., 2005; Ahmad et al., 2010; Jiang et al., 2013). Adding to the complexity, each of these receptors has their own distinct signaling pathways, tissue distribution, and expression profiles (Candelario-Jalil et al., 2005; Johansson et al., 2013). Here, we focus on the role of the EP receptor subtype 2 (EP2), a G protein-coupled receptor that stimulates adenylyl cyclase leading to increases in cytosolic cyclic AMP (cAMP), which in turn engages protein kinase A (PKA) and the exchange protein activated by cAMP (Epac). and Epac signaling are intimately connected and may result in synergistic or opposite effects (Bos, 2006). The temporal gradient of cAMP levels likely determines which pathway is preferentially activated: with early EP2 activation, and thus low cAMP, the PKA pathway presumably predominates, given its higher affinity for cAMP, while the Epac pathway may succeed with continued EP2 activation and rises in cytosolic cAMP (Jiang and Dingledine, 2013). In the brain, PGE_2_-EP2-cAMP-mediated PKA and Epac activation is generally associated with neuroprotective and neurotoxic effects, respectively (McCullough et al., 2004; Suzuki et al., 2010; Hara et al., 2012; Mohan et al., 2015).

Using *in vitro* and *in vivo* models and genetic and pharmacologic approaches, the role of the EP2 receptor in mediating outcomes of various neurological conditions has been extensively studied. We, and others, have shown the beneficial effects of EP2 receptor signaling following transient ischemic stroke, permanent focal ischemia, and *N*-Methyl-d-aspartate-induced excitotoxicity (McCullough et al., 2004; Liu et al., 2005; Ahmad et al., 2006b; Ahmad et al., 2010). In contrast, EP2 receptor signaling resulted in worse outcomes, with more oxidative stress and inflammation, in mouse models of Alzheimer’s disease (AD), Parkinson’s disease (PD), amyotrophic lateral sclerosis (ALS), and epilepsy (Liang et al., 2005; Jin et al., 2007; Liang et al., 2008; Jiang et al., 2012, 2013). All of these conditions have a considerable inflammatory component and collectively these studies suggest that EP2 receptor signaling can have either positive or negative immunomodulatory effects depending on the underlying neuropathological process.

Given the importance of PGE_2_-EP2 receptor signaling in a wide range of acute and chronic neurological conditions, we sought to determine the respective and unique contribution of EP2 in modulating ICH-induced brain injury and inflammation. We found that genetic deletion of the EP2 receptor results in improved anatomical and functional recovery following ICH and is associated with less blood and ferric iron accumulation, neuroinflammation, blood–brain barrier (BBB) breakdown, and peripheral neutrophil infiltration. The PGE_2_ EP2 receptor appears to play an important role in modulating ICH-induced brain injury and inflammation and could represent a new therapeutic target for the treatment of this condition, which currently has no effective therapies.

## Materials and Methods

### Mice

Studies were performed on 2.5- to 5-month-old male wildtype (WT, 3.2 ± 0.8 months, *n* = 8) and EP2 receptor knockout (EP2^−/−^, 4.5 ± 0.5 months, *n* = 12) C57BL/6 mice. The EP2^−/−^ mice develop normally, gain weight at a rate equal to that of WT animals, and have no gross anatomical or behavioral abnormalities when compared with WT littermates (Hizaki et al., 1999; Ahmad et al., 2006b). Prior to experiments, the genotype of EP2^−/−^ mice was confirmed by polymerase chain reaction. Colonies were maintained in our animal facilities in a temperature-controlled environment (23 ± 2℃) on a 12 hr reverse dark/light cycle, so behavioral testing could be performed during the awaken phase. Mice were allowed ad libitum access to food and water before and after surgical procedures. The Institutional Animal Care and Use Committee at the University of Florida approved all animal protocols.

### ICH Model

ICH was induced in WT and EP2^−/−^ mice as described previously with modifications (Chen et al., 2011; Singh et al., 2013). Additional changes were incorporated in order to avoid needle insertion through the motor cortex and thus the possibility of confounding results on behavioral analyses and to improve the modeling of clinical deep basal ganglia hemorrhages, where concomitant intraventricular hemorrhage (IVH) is seen in 40% of nontraumatic ICH cases and is associated with poor long-term prognosis (Moradiya et al., 2014; Poon et al., 2014). Changes were accomplished by modifying the site and angle of craniotomy/needle insertion and the site of injection within the striatum. Briefly, stereotactic equipment was first manipulated so the injection could be performed into the left hemisphere at a 40° angle from the vertical plane. Mice were anesthetized using isoflurane (4% induction, 1.5–2% maintenance) and immobilized on a stereotactic frame (Stoelting, Wood Dale, IL). A small left-sided incision in the skin overlying the skull was made in a coronal plane midway between the left eye and ear. A craniotomy was performed at an angle matching that of the stereotactic angle at the following coordinates: 0.0 mm anteroposterior and 3.8 mm left, relative to bregma. A syringe with a 26-gauge needle (Hamilton Co., Reno, NV) was inserted 3.6 mm ventral from the skull surface and 0.04 units of collagenase type VII-S (Sigma, St. Louis, MO) dissolved in 0.40 µl of sterile water was infused at 0.20 µl/min using an automated injector (Stoelting). The needle was left in place for 5 min and then slowly removed over a 15 min period. Rectal temperatures were maintained at 37.0 ± 0.5℃ throughout all surgical procedures and mice were allowed to fully recover in temperature and humidity-controlled chambers postoperatively.

### Determination of Initial Hemorrhage Volume

As different initial hemorrhage volumes in WT and EP2^−/−^ mice after collagenase-induced ICH could influence anatomical and functional recovery, we assessed brain hemoglobin content in both groups 5 hr as we have described (Wang and Doré, 2007a). Briefly, WT (*n* = 3) and EP2^−/−^ (*n* = 4) mice were deeply anesthetized and transcardially perfused with phosphate-buffered saline (PBS). After quick removal of the brain, the olfactory bulbs and cerebellum were discarded and the ipsilateral and contralateral hemispheres were separately snap frozen in 2-methylbutane precooled over dry ice. Samples were thawed and homogenized for 5 min in 700 µl of sterile deionized water. After centrifugation at 14,000 rpm and 4℃ for 30 min, the supernatant was used for hemoglobin determination by Drabkin’s method (Sigma, St. Louis, MO). An eight-point standard curve was generated by spiking 15 µl of lysed citrate anticoagulated blood, collected by intracardial puncture from control animals, into 135 µl of a pooled contralateral hemisphere supernatant with twofold dilutions thereafter. In a 96-well plate, 50 µl of Drabkin’s reagent was added to 50 µl of supernatant. All samples were run in triplicate. After 15 min incubation at room temperature, cyanmethemoglobin concentration, reflecting brain hemoglobin content and thus initial hemorrhage volume, was determined by absorbance at 540 nm.

### Functional Outcomes

Functional outcomes were assessed daily post-ICH by neurological deficit scoring (NDS), accelerating rotarod performance, and open field locomotor activity. Testing was performed during the dark cycle (awaken phase) by investigators blinded to genotype. Each test was performed at the same time of the day and mice were allowed 1 hr of rest in between tests. NDS: two blinded investigators independently assessed mice for deficits in neurological functioning as we have described (Glushakov et al., 2013). Accelerating rotarod performance: mice were evaluated for motor deficits and coordination, endurance, and balance using an accelerating rotarod Rotamex-5 machine and software (Columbus, OH). Rotational speed started at 4 rpm and ended at 30 rpm, and the latency to fall was automatically collected by the software. On the three consecutive days prior to surgery, mice were trained twice per day (morning and late afternoon), with three cycles per training period. Average performance on the sixth training period served as baseline functioning. Post-ICH testing consisted of one testing period per day with three cycles and data are reported as the average latency to fall. Open field locomotor activity: ambulatory distance and stereotypic time were measured using an automated open field activity monitor and video tracking interface system (MED associates, St. Albans, VT). Baseline locomotor activity was assessed the day prior to surgery, before rotarod training and pretesting. Briefly, mice were placed individually in four transparent acrylic cages and their locomotor activity was recorded over a 30-min test period. The first 5 min of recorded data was omitted to exclude for initial anxiety responses.

### Histology

At 72 hr post-ICH, mice were deeply anesthetized and transcardially perfused with PBS (pH 7.4) followed by 4% paraformaldehyde (PFA). Brains were collected and kept in 4% PFA for at least 24 hr prior to cryopreservation in a 30% sucrose/PBS solution. Ten sets of 16 sections equally distributed throughout the entire hematoma and anteroposterior brain regions were processed at 30 µm and stored at −80℃ for later histological procedures. In this way, for each animal, multiple staining procedures can be performed and the staining pattern throughout the whole brain can be analyzed. Cresyl Violet staining was used to assess lesion volume, ipsilateral hemispheric enlargement, blood accumulation, and the incidence of IVH (Ahmad et al., 2006a). To estimate the ferric iron content, Perls’ iron stain was completed by incubating slides in a 1:1 mix of 2% hydrochloric acid and 2% potassium ferrocyanide for 20 min, followed by counterstaining with nuclear fast red. Immunohistochemistry was performed to evaluate microgliosis, astrogliosis, BBB breakdown, and neutrophil infiltration using the following primary antibodies: ionized calcium-binding adapter protein 1 (Iba1), 1:1,000 (Wako, Richmond, VA); glial fibrillary acidic protein (GFAP), 1:1,000 (Dako, Carpinteria, CA); immunoglobulin G (IgG), 1:300 (Vector Laboratories, Burlingame, CA); and myeloperoxidase (MPO), 1:500 (Pierce, Dallas, TX), respectively. A secondary biotinylated antibody was used for detection (Vector Laboratories), except for in the case of IgG staining, which used a biotinylated primary anti-mouse IgG antibody. The Vectastain Elite Avidin/biotinylated enzyme complex and 3,3′-diaminobenzidine (DAB) kits (Vector Laboratories) were used per manufacturer’s instructions for the avidin-peroxidase step and final DAB reaction, respectively. MPO and IgG slides were counterstained with Cresyl Violet, while GFAP and Iba1 slides were not counterstained. After Cresyl violet, Perls’ iron, and immunohistochemistry, slides were dehydrated in increasing concentrations of ethanol and coverslipped with Permount.

### Quantification Procedures

All slides were scanned using a ScanScope CS and analyzed with ImageScope software (Aperio Technologies, Inc., Vista, CA). For quantification procedures in which total brain pathology was analyzed (lesion volume, ipsilateral hemispheric enlargement, blood accumulation, ferric iron content, BBB breakdown, and peripheral neutrophil infiltration), all 16 sections were quantified for each animal. To assess astrogliosis and microgliosis, the same four sections representing maximal lesion area for each animal were analyzed. Lesion volume: injured brain regions were outlined, areas abstracted from the ImageScope software, and a volume was calculated using these areas, known distance between each section, and section thickness. Percentage of ipsilateral hemispheric enlargement: the whole brain and contralateral hemisphere were outlined, volumes determined as described above, and the percentage of ipsilateral hemispheric enlargement was calculated by 100 × [(ipsilateral-contralateral)/contralateral]. IVH: the visual presence of red blood cells (RBCs) within the lateral ventricles was counted as IVH. Ferric iron content and immunohistochemical stains: the ImageScope Positive Pixel Count software was used for quantification after the appropriate brain regions were outlined (see below). Algorithms were developed for each of the stains such that the appropriate signal and strength of signal were evaluated (e.g. blue pixels in Perls’ stained slides). These algorithms were tuned so moderate and strongly positive pixels were detected and weakly positive pixels were excluded as these could potentially represent nonspecific signal. Cortical microgliosis was analyzed by placing identically sized boxes of 1,000 by 1,000 pixels in the ipsilateral and contralateral motor cortex. Data are presented as the relative ipsilateral to contralateral signal. Striatal microgliosis was analyzed by outlining of the ipsilateral and contralateral striatum, excluding the lesion area. Data are presented as the ipsilateral signal per area quantified, with normalization for the contralateral signal per area quantified. Cortical astrogliosis and striatal astrogliosis were analyzed in a similar manner to microgliosis; however, these data were not normalized for the contralateral equivalent due to negligible signal in the contralateral cortex and striatum with the thresholds used here. Blood accumulation, ferric iron content, and MPO slides were analyzed by circling of the ipsilateral hemisphere. Whole brain signal was examined for IgG quantification as staining extended into the contralateral side in some cases. After all analyses, the appropriate algorithm was run and signal data were abstracted from the ImageScope software. As total ferric iron content, BBB breakdown, and neutrophil infiltration were analyzed, the values for each animal were individually corrected for lesion volume such that corrected and uncorrected data are presented.

### Statistics

GraphPad Prism 6 software was used for all statistical analyses. Differences between two groups were determined by an unpaired two-tailed parametric Student’s *t*-test. The incidence rate for IVH was compared using Fisher’s exact test. Behavioral data were analyzed using a repeated measures two-way analysis of variance and Newman–Keuls multiple comparisons test. Data are expressed as mean ± SEM and *p* < .05 was considered statistically significant in all analyses.

## Results

Following induction of ICH in WT and EP2^−/−^ mice, consistent striatal hemorrhages were seen in both groups at 72 hr postictus and various anatomical and functional outcomes were evaluated by histology and neurobehavioral testing, respectively. There were no significant differences in percent body weight loss at any time point post-ICH between the groups. The mortality rates were 12.5% (1 out of 8) and 8.3% (1 out of 12) for the WT and EP2^−/−^ mice, respectively.

### EP2 Receptor Deletion Reduces Brain Injury After ICH

**Figure 1.**
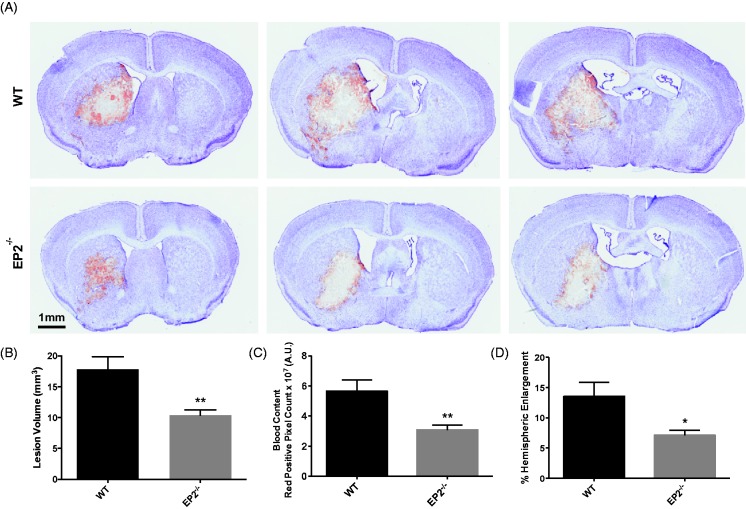
Genetic deletion of the PGE_2_ EP2 receptor reduces brain injury after ICH. WT and EP2^−/−^ mice underwent ICH and were sacrificed at 72 hr for determination of lesion volume by Cresyl violet staining of brain sections. (a) Representative images of coronal brain sections from WT (upper panels) and EP2^−/−^ mice (lower panels). Images are from the same animal and demonstrate characteristic hematoma profiles, where left to right corresponds to anterior to posterior. Center images are adjacent to the needle insertion site and represent maximal hematoma size. (b) Quantification of lesion volumes showed that EP2^−/−^ mice had significantly less ICH-induced brain injury. (c) Red/brown positive pixel count analysis demonstrated that EP2^−/−^ mice had significantly less blood accumulation within the injured brain area. (d) EP2^−/−^ mice had less percentage of ipsilateral hemispheric enlargement. *p < .05 and **p < .01, all comparisons included *n* = 7 WT and *n* = 10 EP2^−/−^ mice. PGE_2_ = prostaglandin E_2_; EP2 = E prostanoid receptor subtype 2; ICH = intracerebral hemorrhage; WT = wildtype.

When compared with WT controls, EP2^−/−^ mice displayed significantly reduced striatal lesions associated with less blood accumulation and ipsilateral hemispheric enlargement (Figure 1(a)). Quantification of lesion volume showed that EP2^−/−^ mice had 41.9 ± 4.7% smaller lesions when compared with WT controls (10.4 ± 0.8 mm^3^ vs. 17.8 ± 2.0 mm^3^, *p* = .0015, Figure 1(b)). As identified by red/brown positive pixel count, the EP2^−/−^ mice had 45.5 ± 5.8% less blood accumulation (3.1 ± 0.3 × 10^7 ^A.U. vs. 5.7 ± 0.8 × 10^7 ^A.U., *p* = .0032, Figure 1(c)). EP2^−/−^ mice also displayed 47.4 ± 6.3% less percent ipsilateral hemispheric enlargement (7.1 ± 0.9% vs. 13.5 ± 2.4%, *p* = .0111, Figure 1(d)). IVH occurred in 53% of the mice, although EP2^−/−^ mice had a significantly decreased incidence of IVH when compared with WT controls (20% vs. 100%, *p* = .0023). The presence of IVH was associated with an enlargement of the lateral ventricles (Figure 1(a)).

### EP2 Receptor Deletion Has No Effect on Collagenase-Induced Bleeding

Initial brain hemoglobin levels were measured at 5 hr post-surgery to determine if the improved outcomes in EP2^−/−^ mice resulted from differences in collagenase-induced bleeding between the genotypes. Brain hemoglobin content was not significantly different between WT and EP2^−/−^ mice (EP2^−/−^: 0.092 ± 0.010 A.U., WT: 0.102 ± 0.011 A.U., *p* = .5142), indicating no differences in susceptibility to collagenase-induced bleeding and initial hemorrhage volume.

### Effect of EP2 Receptor Deletion on Functional Outcomes After ICH

**Figure 2.**
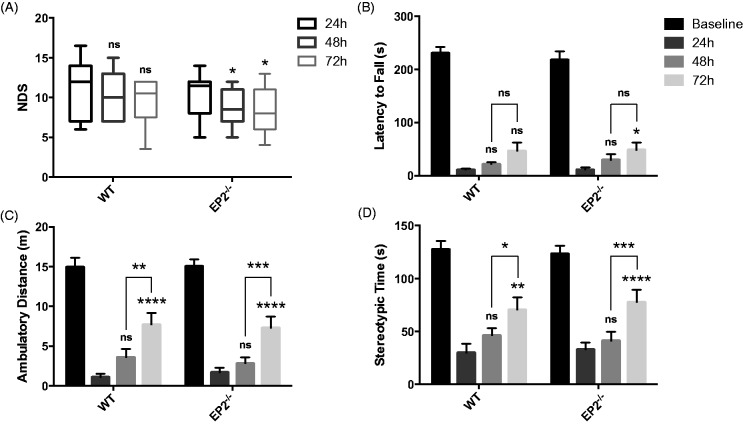
Effect of PGE_2_ EP2 receptor deletion on functional outcomes after ICH. Neurobehavioral testing of WT and EP2^−/−^ mice was performed by investigators blinded to genotype at 24, 48, and 72 hr following ICH. (a) At 48 and 72 hr after ICH, EP2^−/−^ mice had significantly less neurological deficits when compared with 24 hr, whereas no such recovery was seen for the WT mice. (b) WT and EP2^−/−^ mice had similar baseline rotarod performance. At 24, 48, and 72 hr after ICH, both groups had significantly reduced latency to fall when compared with baseline function (*p* < .0001). At 72 hr after ICH, EP2^−/−^ mice had improved accelerating rotarod performance when compared with 24 hr, whereas this comparison was not significant for the WT mice. (c and d) No differences in baseline open field locomotor activity were seen for the WT and EP2^−/−^ mice. Both groups had significantly impaired (c) ambulatory distance and (d) stereotypic time at 24, 48, and 72 hr post-ICH when compared with baseline function (*p* < .0001). WT and EP2^−/−^ mice displayed similar open field locomotor activity, where significant improvements in (c) ambulatory distance and (d) stereotypic time were seen between 48 and 72 hr post-ICH, but not within the 24- to 48-hr period. All comparisons included *n* = 7 WT and *n* = 11 EP2^−/−^ mice, and statistics were calculated using a two-way repeated measures analysis of variance with Newman–Keuls multiple comparisons test. Brackets outline 48 to 72 hr comparisons and results above bars are with respect to 24 hr function. ns = not significant, *p < .05. **p < .01. ***p < .001. ****p < .0001. PGE_2_ = prostaglandin E_2_; EP2 = E prostanoid receptor subtype 2; ICH = intracerebral hemorrhage; WT = wildtype.

Functional outcomes were assessed by several neurobehavioral tests performed by investigators blinded to genotype at 24, 48, and 72 hr after ICH. For all tests, including NDS, accelerating rotarod performance, and open field locomotor activity, we did not find significant differences between the WT and EP2^−/−^ groups at any time point post-ICH. WT and EP2^−/−^ mice performed similarly on baseline testing for rotarod and open field locomotor activity. At 24, 48, and 72 hr after ICH, both groups performed worse on the rotarod and had reduced ambulatory and stereotypic time when compared with baseline function (*p* < .0001). EP2^−/−^ mice had less neurologic deficits at 48 and 72 hr after ICH, when compared to 24 hr (*p* < .05 for both time points), whereas no such improvement was seen in the WT group (Figure 2(a)). Similarly, the EP2^−/−^ mice performed better on the rotarod at 72 hr after ICH, when compared to 24 hr (*p* < .05), whereas no significant functional differences were seen for the WT group (Figure 2(b)). WT and EP2^−/−^ mice had similar open field locomotor activity, where significant improvements in ambulatory distance were seen at 72 hr when compared to 24 hr (*p* < .0001 for both WT and EP2^−/−^) and 48 hr (WT: *p* < .01, EP2^−/−^: *p* < .001); whereas, no differences were seen between 24 and 48 hr (Figure 2(c)). Likewise, both groups had significant recovery in stereotypic time at 72 hr after ICH when compared to 24 hr (WT: *p* < .01, EP2^−/−^: *p* < .0001) and 48 hr (WT: *p* < .05, EP2^−/−^: *p* < .001), with no significant differences between 24 and 48 hr (Figure 2(d)). These results indicate that more post-ICH recovery in locomotor activity occurs within the 48- to 72-hr period, with fewer improvements between 24 and 48 hr.

### EP2 Receptor Deletion Reduces Brain Ferric Iron Content After ICH

**Figure 3.**
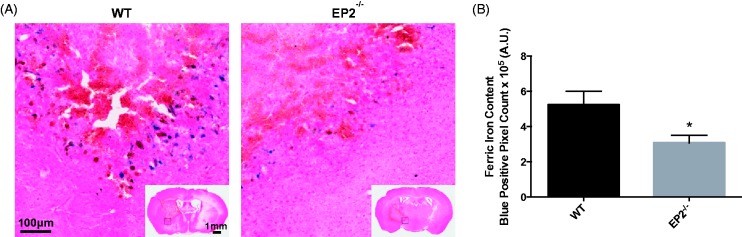
Genetic deletion of the PGE_2_ EP2 receptor reduces brain ferric iron Perls’ staining content after ICH. WT and EP2^−/−^ mice underwent ICH and were sacrificed at 72 hr for determination of ferric iron content by Perls’ staining of brain sections. (a) Representative high magnification images of coronal brain sections showing ferric iron accumulation (blue) in perihematomal regions from WT (left panel) and EP2^−/−^ mice (right panel). Square selections in the inserts denote magnified regions. (b) Quantification of blue positive pixel count in the ipsilateral hemisphere showed that EP2^−/−^ mice had significantly less ferric iron deposition (WT: *n* = 7, EP2^−/−^: *n* = 10, *p < .05). No ferric iron was seen in the contralateral hemisphere for any of the mice in the study. PGE_2_ = prostaglandin E_2_; EP2 = E prostanoid receptor subtype 2; ICH = intracerebral hemorrhage; WT = wildtype.

In order to start addressing potential mechanisms of action, Perls’ iron staining was performed to evaluate brain ferric iron content. In both WT and EP2^−/−^ mice, deposition of ferric iron (blue) was noted primarily in perihematomal regions (Figure 3(a)). Quantification of blue positive pixel count showed that EP2^−/−^ mice had 41.4 ± 8.1% less ferric iron in the ipsilateral hemisphere when compared with WT controls (3.1 ± 0.4 × 10^5 ^A.U. vs. 5.2 ± 0.8 × 10^5 ^A.U., *p* = .0186, Figure 3(b)). No ferric iron was seen in the contralateral hemisphere for any of the mice in the study. When the ferric iron content was corrected for animal lesion volume, significance was lost (EP2^−/−^: 3.1 ± 0.5 × 10^4 ^A.U. per mm^3^, WT: 3.3 ± 0.7 × 10^4 ^A.U. per mm^3^, *p* = .8351).

### Effect of EP2 Receptor Deletion on Microglial Activation and Astrogliosis After ICH

**Figure 4.**
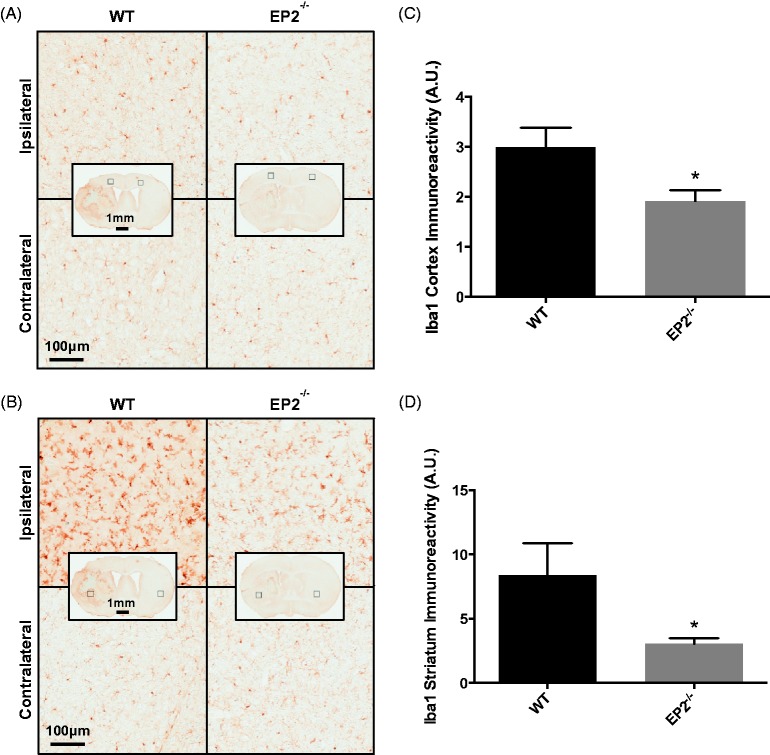
Effect of PGE_2_ EP2 receptor deletion on microglial activation after ICH. Seventy-two hours after ICH, WT and EP2^−/−^ mice were sacrificed and brains processed for Iba1 immunohistochemistry to evaluate cortical and striatal microglial activation and morphological changes. (a and b) Representative high magnification images of coronal brain sections showing the ipsilateral and contralateral (a) cortex and (b) striatum for WT (left panels) and EP2^−/−^ mice (right panels). Square selections in the inserts denote magnified regions. Cortical and striatal images are from the same WT and EP2^−/−^ animals. (c and d) Quantification of brown positive pixel count demonstrated that EP2^−/−^ mice had significantly less **(**c**)** cortical and (d) striatal Iba1 immunoreactivity. This reduced microglial activation was accompanied by less morphological changes. All data are normalized to the corresponding contralateral equivalent areas. Comparisons included *n* = 7 WT and *n* = 10 EP2^−/−^ mice, *p < .05. PGE_2_ = prostaglandin E_2_; EP2 = E prostanoid receptor subtype 2; ICH = intracerebral hemorrhage; WT = wildtype; Iba1 = Ionized calcium-binding adapter protein.

Furthermore, microgliosis was evaluated by immunohistochemistry for Iba1. When compared with WT controls, EP2^−/−^ mice had less cortical and striatal microglial activation and morphological changes as a result of the ICH. Both WT and EP2^−/−^ mice displayed significantly increased microglial activation and morphological changes in the ipsilateral cortex (Figure 4(a)) and striatum (Figure 4(b)) compared with the contralateral equivalent areas. After quantification, EP2^−/−^ mice had 36.1 ± 7.5% less cortical microgliosis when compared with WT controls (1.9 ± 0.2 A.U. vs. 3.0 ± 0.4 A.U., *p* = .0220, Figure 4(c)). Similarly, EP2^−/−^ mice had 63.6 ± 5.2% less striatal microgliosis (3.1 ± 0.4 A.U. vs. 8.4 ± 2.5 A.U., *p* = .0244, Figure 4(d)). The increased activation and morphological changes were considerably more pronounced in the ipsilateral striatum when compared with the ipsilateral cortex for both groups (EP2^−/−^: *p* = .0143, WT: *p* = .0491); although, EP2^−/−^ mice experienced less of a relative cortical to striatal increase in microgliosis compared with WT controls. No significant differences in microglial activation or morphological changes were seen between the two genotypes in the contralateral striatum or cortex.

**Figure 5.**
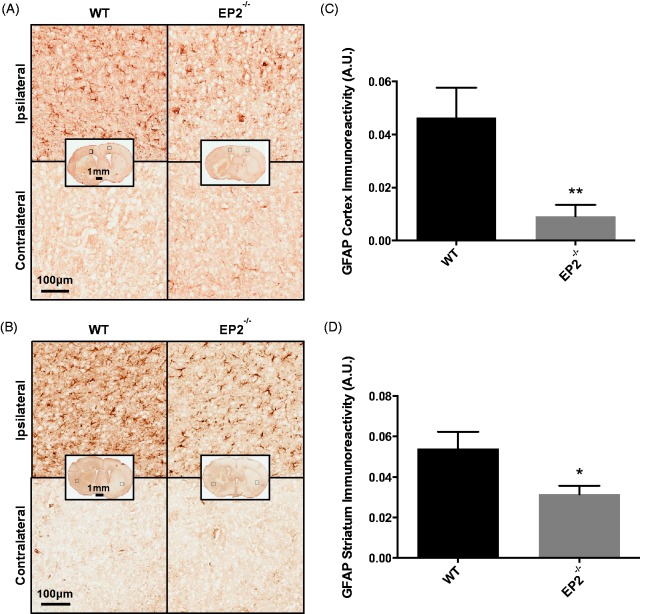
Effect of PGE_2_ EP2 receptor deletion on astrogliosis after ICH. Seventy-two hours after ICH, WT and EP2^−/−^mice were sacrificed and brains processed for GFAP immunohistochemistry to evaluate cortical and striatal astrogliosis. (a and b) Representative high magnification images of coronal brain sections showing the ipsilateral and contralateral (a) cortex and (b) striatum for WT (left panels) and EP2^−/−^ mice (right panels). Square selections in the inserts denote magnified regions. (c and d) Quantification of brown positive pixel count demonstrated that EP2^−/−^ mice had significantly less (c) cortical and (d) striatal GFAP immunoreactivity. Both groups demonstrated negligible staining in the contralateral cortex and striatum; thus, data are presented as ipsilateral immunoreactivity corrected for the area of quantification without normalization for the contralateral equivalent. Comparisons included *n* = 7 WT and *n* = 10 EP2^−/−^ mice, *p < .05, **p < .01. PGE_2_ = prostaglandin E_2_; EP2 = E prostanoid receptor subtype 2; ICH = intracerebral hemorrhage; WT = wildtype; GFAP = glial fibrillary acidic protein.

Astrogliosis was evaluated by immunohistochemistry for GFAP. When compared with WT controls, EP2^−/−^ mice had less cortical and striatal astrogliosis as a result of the ICH. Both WT and EP2^−/−^ mice displayed significantly increased astrogliosis in the ipsilateral cortex (Figure 5(a)) and striatum (Figure 5(b)) compared with the contralateral equivalent areas. After quantification, EP2^−/−^ mice had 80.3 ± 9.3% less cortical astrogliosis when compared with WT controls (0.0091 ± 0.0043 A.U. vs. 0.0465 ± 0.0112 A.U., *p* = .0031, Figure 5(c)). Similarly, EP2^−/−^ mice had 41.8 ± 7.7% less striatal astrogliosis (0.0314 ± 0.0042 A.U. vs. 0.0540 ± 0.0083 A.U., *p* = .0181, Figure 5(d)). The increased astrogliosis was considerably more pronounced in the ipsilateral striatum when compared with the ipsilateral cortex for the EP2^−/−^ mice (*p* = .0016), whereas no significant difference was seen for the WT controls (*p* = .5977), given the relatively strong cortical astrogliosis in this group.

### EP2 Receptor Deletion Reduces Peripheral Neutrophil Infiltration After ICH

**Figure 6.**
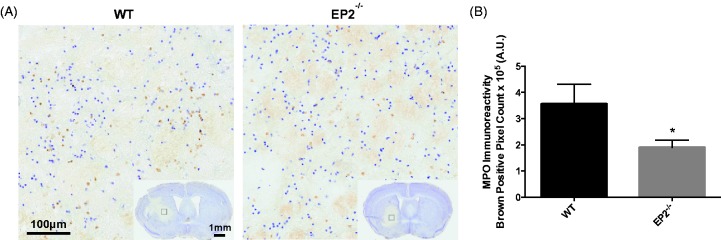
Genetic deletion of the PGE_2_ EP2 receptor reduces peripheral neutrophil infiltration after ICH. Seventy-two hours after ICH, WT and EP2^−/−^ mice were sacrificed and brains processed for MPO immunohistochemistry to evaluate peripheral neutrophil infiltration. (a) Representative high magnification images of coronal brain sections showing diffusely distributed MPO + cells within the lesion. Square selections in the inserts denote magnified regions. (b) Quantification of brown positive pixel count in the ipsilateral hemisphere showed that EP2^−/−^ mice had significantly less peripheral neutrophil infiltration (WT: *n* = 5, EP2^−/−^: *n* = 10, *p < .05). No MPO + cells were seen outside of the injured brain area for any of the mice in the study. PGE_2_ = prostaglandin E_2_; EP2 = E prostanoid receptor subtype 2; ICH = intracerebral hemorrhage; WT = wildtype; MPO = myeloperoxidase.

In addition, immunohistochemical staining for MPO was performed to evaluate peripheral neutrophil infiltration. MPO + cells were diffusely distributed throughout the hematoma, with occasional focal sites of concentration surrounding blood vessels (Figure 6(a)). Quantification of brown positive pixel count showed that EP2^−/−^ mice had 46.8 ± 7.9% less neutrophil infiltration when compared with WT controls (1.9 ± 0.3 × 10^5 ^A.U. vs. 3.6 ± 0.8 × 10^5 ^A.U., *p* = .0239, Figure 6(b)). No MPO + cells were seen outside of the injured brain area for any of the mice in the study. When the MPO signal was corrected for animal lesion volume, significance was lost (EP2^−/−^: 1.9 ± 0.3 10^4 ^A.U. per mm^3^, WT: 1.9 ± 0.3 10^4 ^A.U. per mm^3^, *p* = .9260).

### Effect of EP2 Receptor Deletion on BBB Breakdown After ICH

**Figure 7.**
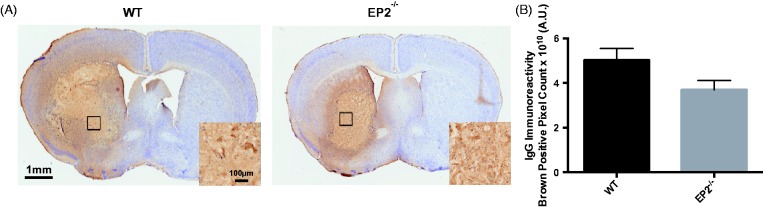
Effect of PGE_2_ EP2 receptor deletion on BBB breakdown after ICH. Seventy-two hours after ICH, WT and EP2^−/−^ mice were sacrificed and brains processed for IgG immunohistochemistry to evaluate BBB breakdown. (a) Representative images of coronal brain sections showing more diffuse disruption of the BBB in WT mice. Square boxes denote the location of high magnification inserts. (b) Quantification of whole brain brown positive pixel count showed that EP2^−/−^ mice strongly tended to have reduced BBB breakdown (WT: *n* = 6, EP2^−/−^: *n* = 10, *p* = .0668). PGE_2_ = prostaglandin E_2_; EP2 = E prostanoid receptor subtype 2; ICH = intracerebral hemorrhage; WT = wildtype; BBB = blood–brain barrier; IgG = immunoglobulin G.

Finally, immunohistochemistry for mouse IgG was performed to evaluate BBB breakdown. WT mice had a more diffuse staining pattern when compared with EP2^−/−^ mice (Figure 7(a)). After quantification of whole brain brown positive pixel count, EP2^−/−^ mice strongly tended to have reduced BBB breakdown by 26.4 ± 8.1% when compared with WT controls (3.7 ± 0.4 × 10^10 ^A.U. vs. 5.0 ± 0.5 × 10^10 ^A.U., *p* = .0668, Figure 7(b)). When the IgG signal was corrected for animal lesion volume, significance was lost (EP2^−/−^: 3.5 ± 0.4 × 10^9 ^A.U. per mm^3^, WT: 3.0 ± 0.2 × 10^9 ^A.U. per mm^3^, *p* = .3566).

## Discussion

This study is novel as it specifically evaluates the respective role of the PGE_2_-EP2 receptor in modulating brain injury and neuroinflammation following ICH. Here, we unveil that genetic deletion of the EP2 receptor results in smaller brain lesion volumes associated with less blood and ferric iron accumulation. EP2^−/−^ mice also displayed less ipsilateral hemispheric enlargement and incidence of IVH. With the neurobehavioral tests employed here, no significant differences were seen when comparing functional outcomes of WT and EP2^−/−^ mice at the same time point post-ICH; however, EP2^−/−^ mice did display improved recovery identified by NDS at 48 and 72 hr. These improvements in anatomical outcomes and functional recovery were accompanied by less cortical and striatal microgliosis, cortical and striatal astrogliosis, BBB breakdown, and peripheral neutrophil infiltration. Together, these data suggest a deleterious role for PGE_2_-EP2 receptor signaling following ICH.

In the brain, the EP2 receptor has been reported to be highly and broadly expressed by neurons and glia in the cerebral cortex, striatum, thalamus, and hippocampus of mice (Zhang and Rivest, 1999; McCullough et al., 2004; Ahmad et al., 2006b; Jiang and Dingledine, 2013; Johansson et al., 2013), where it has been shown to modulate responses to several CNS insults including cerebral ischemia, epilepsy, AD, PD, and ALS (Liang et al., 2005; Liu et al., 2005; Jin et al., 2007; Liang et al., 2008; Ahmad et al., 2010; Jiang et al., 2012, 2013). Here, reduced lesion volumes, blood accumulation, and ferric iron content in EP2^−/−^ mice after ICH suggest a negative role for EP2 receptor signaling in mediating blood clearance. A few studies have looked into the contribution of EP2 in modulating phagocytosis in the context of AD, PD, and antibacterial defense in the lung. Deletion or blockade of the EP2 receptor restored alveolar macrophage phagocytic ability of IgG-opsonized RBCs in response to stimulation with apoptotic cells (Medeiros et al., 2009). Activation of the EP2 receptor by PGE_2_ and butaprost, a selective EP2 agonist, reduced β-amyloid phagocytosis by cultured rat microglia (Nagano et al., 2010). In another study, deletion of the EP2 receptor enhanced microglial clearance of β-amyloid in human brain sections and reduced microglial-mediated β-amyloid paracrine neurotoxicity (Shie et al., 2005a). Similarly, EP2 receptor deletion augmented microglial-mediated clearance of α-synuclein aggregates in the mesocortex of patients with Lewy body disease (Jin et al., 2007). These studies collectively suggest that signaling through the EP2 receptor impairs the phagocytic capabilities of macrophages and microglia, both of which are cell types responsible for clearance of RBCs and debris following ICH. Furthermore, the EP2 receptor is reported to be the most strongly expressed E prostanoid receptor on macrophages (Zaslona et al., 2012). Thus, it is possible that in this study, EP2^−/−^ mice showed smaller ICH-induced brain lesions with less blood and ferric iron accumulation as a result of enhanced phagocytic capability, resulting in improved clearance of RBCs and damaged tissue.

Differences in the vasculature and susceptibility to collagenase-induced bleeding are other possible mechanisms that could lead to smaller lesion volumes in EP2^−/−^ mice following ICH. We have previously shown that genetic deletion of the EP2 receptor does not significantly alter the gross vascular anatomy of the brain (Ahmad et al., 2010). Although we cannot exclude that structural differences exist in the vessels of EP2^−/−^ mice on a microscopic scale, which could alter ICH outcomes, we have shown that the initial bleed volume is not significantly different between the two genotypes. Equivalent initial hemorrhage volumes imply that the underlying anatomical structure of small penetrating striatal vessels is not significantly changed with EP2 receptor deletion and that these mice are equally susceptible to collagenase-induced bleeding. These findings further point toward a blood clearance mechanism as the etiology of improved outcomes in EP2^−/−^ mice after ICH.

The EP2^−/−^ mice also demonstrated reduced ipsilateral hemispheric enlargement and less incidence of IVH, which both may simply be attributed to smaller lesion volumes. However, we cannot rule out that the former may also be a result of EP2-mediated increased neuroinflammation and edema formation in WT mice. Although we have shown the substantially reduced gliosis in EP2^−/−^ mice after ICH, additional studies would be needed to determine whether edema is participating in the increased ipsilateral hemispheric enlargement and whether this edema was directly as a result of signaling through the EP2 receptor. Whereas the decreased incidence of IVH in EP2^−/−^ mice is most likely as a result of smaller lesion volumes and less extension of blood into the lateral ventricles. It should be noted that the modifications we incorporated into the commonly used ICH model employed in this study (see Methods) resulted in approximately the same overall incidence of IVH after ICH as is seen clinically (Bhattathiri et al., 2006; Moradiya et al., 2014; Poon et al., 2014). Furthermore, the presence of IVH was accompanied by hydrocephalus (e.g., dilation or enlargement of the lateral ventricles) as occurs in patients with IVH alone or who have IVH as a result of a primary ICH or other type of acute brain damage (Bhattathiri et al., 2006). Therefore, this modified ICH model offers two advantages: (a) it results in a more clinically relevant scenario and (b) it avoids any potential confounding effects on neurobehavioral testing because the needle used for injection is not inserted through the motor cortex.

Functional outcomes were not significantly different between the WT and EP2^−/−^ mice on any of the neurobehavioral tests when comparing between these two groups at each testing time point post-ICH. The discrepancy in correlation between anatomical and functional outcomes could be due to the inherent difficulty of detecting subtle differences in neurological function in mice. Moreover, differences in functional outcomes may be exaggerated and easier to detect at later time points with this ICH model given the extensive damage produced early on. Under physiological conditions, PGE_2_-EP2 signaling supports hippocampal-dependent memory formation and synaptic plasticity and EP2^−/−^ mice have been shown to have impairments in these processes (Savonenko et al., 2009; Yang et al., 2009; Furuyashiki and Narumiya, 2011). Thus, another possible reason for the discrepancy could be due to impaired cognition with EP2 receptor deletion, which could negate the positive effects of absent EP2 signaling on motor recovery. Nonetheless, EP2^−/−^ mice did display less neurological deficits at 48 and 72 hr when compared with their 24 hr functioning as identified by NDS, whereas the WT mice did not, suggesting that deletion of the EP2 receptor improves the rate of neurological recovery.

Microglia and astrocytes are strongly activated following ICH, where they dynamically interact to modulate inflammatory responses and neuronal survival/injury. For example, astrocytes can directly mediate neuronal survival through production of neurotrophic and angiogenic factors, modulation of neuronal sensitivity to glutamate toxicity and oxidative stress, and downregulation of microglial secretion of proinflammatory mediators and reactive oxygen species (ROS; Wang and Doré, 2007b). However, with excessive activation, astrocytes can also induce edema, provoke inflammation, and release cytotoxins after ICH (Munakata et al., 2013). Likewise, microglia also have dual roles, where they are protective in their cleanup of RBCs and tissue debris but toxic in their release of ROS and proinflammatory mediators. Deletion of the EP2 receptor resulted in significantly reduced brain gliosis after ICH, with less cortical and striatal microgliosis and astrogliosis. These results are consistent with other studies, which have shown that following various CNS insults, presence of the EP2 receptor contributes to microglial and astrocyte activation and significantly increases the levels of many proinflammatory mediators (Jiang et al., 2013; Johansson et al., 2013; Quan et al., 2013). Furthermore, in a few models, neurotoxicity was associated with microglial-specific EP2 expression (Shie et al., 2005a, 2005b; Johansson et al., 2013), suggesting that EP2-mediated microglial activation and release of proinflammatory mediators from these cells contribute to neuronal injury. An unbiased microarray of microglia derived from mice with EP2 conditionally deleted on myeloid cells revealed a significant reduction in the expression of genes associated with cytokine and chemokine signaling, chemotaxis and cell adhesion, immune cell activation, and cell cycle/mitosis, implying an overall decreased inflammatory and proliferative state of EP2 conditional knockout microglia (Johansson et al., 2013). Of note, COX-2 expression was also highly suppressed. In the context of the current study, this point would mean a deleterious positive feedback loop exists in the WT mice, where EP2 activation leads to increased COX-2 expression, PGE_2_ production, EP2-mediated microglial activation, and proinflammatory responses (Johansson et al., 2013). Collectively, these results broadly imply that PGE_2_ signaling through EP2 receptors promotes brain inflammation, leading to microglial-mediated neuronal injury and suggests that the overall reduced activation state in the absence of EP2 may be responsible for the improved ICH outcomes seen here.

This overall blunted neuroinflammatory response in EP2^−/−^ mice after ICH could also explain the reduced BBB breakdown and peripheral neutrophil infiltration. In addition to COX-2 suppression, IL-1β, CXCR2, and MMP-9 are some of the most downregulated genes in myeloid-specific EP2 conditional knockout mice (Johansson et al., 2013). Also importantly, this conditional knockout has equivalent attenuation of plasma TNFα and IL-6 levels as seen in the global knockout (Johansson et al., 2013), further suggesting the importance of myeloid-specific EP2 signaling in promoting the inflammatory response. CXCR2 is the main receptor responsible for neutrophil migration to sites of inflammation. MMP-9 is known to actively participate in BBB breakdown and increases capillary permeability, thereby contributing to brain edema secondary to hemorrhagic brain injury (Wang and Doré, 2007b). The suppressed MMP-9 expression from microglia devoid of EP2 would also likely be accompanied by less activation of MMP-9 due to suppression of ROS and protease production from reduced numbers of infiltrating neutrophils and comparatively less activated microglia. Injection of recombinant IL-1β into the striatum of rats has been shown to cause a transient neutrophil-dependent increase in the permeability of the BBB (Blamire et al., 2000). Collectively, the reduced expression of these proinflammatory mediators in EP2^−/−^ mice could explain the reduced BBB breakdown and peripheral neutrophil infiltration. Furthermore, another related neurotoxic positive feedback mechanism probably exists, in addition to the one described earlier, as IL-1β is known to increase COX-2 expression leading to even higher levels of PGE_2_, further potentiating the microglial-mediated neuronal injury. Moreover, CXCR2 and MMP-9 may then be upregulated as these are partially controlled by COX-2.

Given that we analyzed total brain ferric iron content, BBB breakdown, and neutrophil infiltration in this study, an attempt was made to determine whether the differences seen in these parameters between WT and EP2^−/−^ mice were mediated by EP2 signaling or as a result of differences in ICH-induced lesion volumes and blood accumulation. The values obtained for each parameter and animal were individually normalized to lesion volume. In this way, uncorrected and corrected results can be compared between WT and EP2^−/−^ groups, where if the same trends are seen in both cases, it suggests a direct effect of EP2 signaling rather than a blood-mediated event. Here, we have shown that after individual normalization for lesion volume, ferric iron content, BBB breakdown, and neutrophil infiltration all became nonsignificant between WT and EP2^−/−^ groups, implying that the improved outcomes in EP2^−/−^ mice resulted from less blood accumulation and associated brain damage, further suggesting a possible primary role of EP2 in negatively modulating microglial/macrophage phagocytosis after ICH. However, additional focused confirmatory studies would be needed in order to validate these indirect analyses implying a blood clearance mechanism and assess the complex role of inflammatory mediators. These additional studies should also incorporate several other approaches to further clarify the role of the PGE_2_-EP2 signaling axis in modulating ICH outcomes and to begin assessing the clinical utility of targeting the EP2 receptor as a possible treatment for ICH, including the following: (a) additional end points, (b) extension to the autologous blood ICH model to address the potential contribution of a collagenase-induced inflammatory response, and (c) pharmacologic manipulation of the EP2 receptor with the currently available selective agonists and antagonists. The latter studies should also assess the safety and efficacy of such a pharmacologic approach with a comprehensive evaluation of the possible side effects on other organs or find a clinically relevant way to acutely block EP2 locally within the brain.

Collectively, this study reveals a likely deleterious role for the PGE_2_-EP2 signaling axis after ICH. We have shown that genetic deletion of the EP2 receptor results in an overall improvement in ICH-induced brain injury, where EP2^−/−^ mice have smaller lesion volumes associated with less blood and ferric iron accumulation and reduced gliosis, BBB breakdown, and peripheral neutrophil infiltration. Thus, modulation of the PGE_2_-EP2 signaling axis may represent a new therapeutic avenue for the treatment of ICH.

## Summary Statement

This study investigates the contribution of the PGE_2_-EP2 signaling axis in modulating intracerebral hemorrhage outcomes. Deletion of the EP2 receptor significantly improved anatomical and functional recovery and reduced gliosis, blood–brain barrier breakdown, and peripheral neutrophil infiltration.
